# Iron Deficiency Anemia in Adult Onset Still's Disease with a Serum Ferritin of 26,387 *μ*g/L

**DOI:** 10.1155/2011/184748

**Published:** 2011-05-12

**Authors:** Sheetal Patel, Seyed Monemian, Ayesha Khalid, Harvey Dosik

**Affiliations:** ^1^Department of Internal Medicine, New York Methodist Hospital, 506 Sixth Street, Brooklyn, NY 11215, USA; ^2^Division of Hematology-Oncology, Department of Internal Medicine, New York Methodist Hospital, 506 Sixth Street, Brooklyn, NY 11215, USA; ^3^Division of Hematology, Department of Internal Medicine, New York Methodist Hospital, 506 Sixth Street, Brooklyn, NY 11215, USA; ^4^Weill Cornell Medical College of Cornell University, 1300 York Avenue, New York, NY 10065, USA

## Abstract

Serum ferritin rises in the anemia of chronic inflammation reflecting increased iron storage and other changes mediated by inflammation. When iron deficiency coexists, the ferritin may not always decline into the subnormal range. We describe the rare interaction of iron deficiency with the extreme hyperferritinemia characteristic of adult onset Still's disease. The combination has clinical relevance and allows deductions about the presence of serum ferritin at 26,387 *μ*g/L despite obvious iron depletion. The diagnosis of iron deficiency anemia was delayed and became fully obvious when her Still's disease remitted and serum ferritin decreased to 6.5 *μ*g/L. The coexistence of iron deficiency should be considered when evaluating a patient with anemia of chronic inflammation even when the ferritin level is elevated several hundredfold. Further insights on ferritin metabolism in Still's disease are suggested by the likelihood that the patient's massive hyperferritinemia in the acute phase of Still's disease was almost entirely of the iron-free apoferritin form.

## 1. Introduction

In the anemia of chronic inflammation, iron metabolism is disturbed and the serum ferritin usually rises moderately, achieving mean levels such as 300–400 *μ*g/L [1]. In this setting, it has become clear that ferritin criteria often must be relaxed to allow the recognition of coexisting iron deficiency in patients who have chronic inflammation [2, 3]. Indeed, ferritin levels less than 100 *μ*g/L may be viewed with suspicion in a patient with inflammatory disease [4]. We describe an extreme manifestation of this situation in a patient with the massive hyperferritinemia of adult onset Still's disease. This illustrates the need for diagnostic flexibility in special disorders, and it provides further insight into the likelihood that ferritin metabolism is both qualitatively and quantitatively disturbed in Still's disease to an unusual extent.

## 2. Case Report

A 38-year-old Haitian woman was admitted to the hospital with a five-day history of evanescent salmon-colored maculopapular rash, generalized fatigue, and joint stiffness worsening. Associated symptoms included high spiking fevers, nausea, vomiting, and decreased appetite. She denied weight loss, infectious contacts, or recent travel. 

Two weeks earlier, she had visited our emergency department (ED) twice. On the first visit, she had a similar salmon-colored rash on her neck, legs, and forearm and was given diphenhydramine. Several days later, she visited the ED because of generalized fatigue and joint stiffness, mainly involving both knees, elbows, and wrists. She was again discharged receiving acetaminophen this time. The patient's joint stiffness, generalized fatigue, and rash persisted, however. Additionally, she developed very high fevers, which now mandated her admission to the hospital. 

Her past history was otherwise unremarkable. However, she had also been having heavy menstrual periods, averaging 7 days per cycle for the past several years. She described a craving for cornstarch and clay since her last pregnancy 5 years ago. She denied blood in her stool and had never been tested for parasites while in Haiti. There was no family history of anemia or bleeding disorders. 

Her physical examination revealed posterior cervical lymphadenopathy and a maculopapular rash involving the neck. Furthermore, warmth and tenderness of the wrist, elbows, and ankle joints were appreciated. The stool was negative for occult blood. 

Pertinent laboratory values on admission were a low hemoglobin (Hgb) (10.3 g/dL), low MCV (67 fl), normal platelet count (272 k/uL), mildly elevated white blood cell count (10.3 k/uL), high RDW (18.3%), and low absolute reticulocyte count (14.2 k/uL). C-reactive protein (139 mg/L) and erythrocyte sedimentation rate (79 mm/hr) were markedly elevated. A comprehensive metabolic panel was normal except for marginally elevated serum aspartate transaminase (72 u/L). Antinuclear antibody (ANA) and rheumatoid factor (RF) were negative. An extensive workup for rheumatologic and infectious processes was done and found to be negative. The serum ferritin level was found to be 26,387 *μ*g/L (normal 13–150 *μ*g/L). Serum iron was 49 *μ*g/dL, total iron binding capacity 215 *μ*g/dL, and iron saturation 23%.

The patient's high grade fevers with persistent rash led to a diagnosis of adult onset Still's disease by Yamaguchi's criteria [5], and her marked hyperferritinemia was compatible with the diagnosis. Subsequently, she was given intravenous methylprednisolone with good response. As her fever subsided, the serum ferritin level initially declined steeply within 6 days and then steadily decreased (Table 1). Her anemia at that time was attributed to her inflammation. She was discharged with tapering doses of oral prednisone.

At her scheduled visit to a hematologist six months later, there was complete resolution of her inflammatory symptoms. Her laboratory data now showed a serum ferritin of 6.5 ng/mL (Table 1). Her Hgb was 9.2 g/dL, MCV 70 fl, serum iron 14 ug/dL, total iron binding capacity 306 ug/dL, and iron saturation 5%. A hemoglobin electrophoresis was normal (Hgb A 97.5%, Hgb A2 2.5%) excluding *β*-thalassemia. These laboratory values now indicated obvious iron deficiency anemia, for which she was subsequently treated with ferrous sulfate 325 mg three times a day. When seen 4 months later after starting iron therapy, her blood count and iron studies had returned to normal and her pica resolved (Table 1).

## 3. Discussion

Anemia of chronic inflammation features a network of intracellular absorptive and plasma iron changes that are mediated by hepcidin. Ferritin is often moderately elevated (Table 2) reflecting the fact that increased iron is retained in intracellular stores causing serum ferritin to rise as an acute phase reactant [6–9]. In the presence of coexisting iron deficiency anemia, the serum ferritin often remains modestly elevated despite the iron depletion. In the setting of anemia of chronic inflammation, extra leeway is often allowed when assessing serum ferritin levels if a diagnosis of superimposed iron deficiency anemia is suspected [10]. 

Our patient is unique because of a heretofore undescribed partial masking of coexisting iron deficiency anemia by the extreme hyperferritinemia which is typical for adult onset Still's disease [11]. Although multiple biomarkers such as C-reactive protein reflect the systemic inflammatory nature of Still's disease, none are specific for it. Serum ferritin is considered a useful diagnostic and disease activity marker for Still's disease, however. The serum ferritin levels are usually higher than in any other autoimmune or inflammatory disease and very high levels between 3,000–30,000 *μ*g/L are not uncommon (Table 2) [12]. A cutoff for ferritin levels of 1,000 *μ*g/L has been used to indicate Still's disease in many studies [12, 13].

Despite the extreme initial hyperferritinemia of 26,387 *μ*/L, iron deficiency anemia was eventually recognized in our patient. Her iron-deficiency anemia had likely been present for many years as she described pica (which is not unusual in iron deficient women) for the past 5 years. A longstanding microcytosis, with an MCV as low as 67, anisocytosis with a high RDW, and a history of menorrhagia, give further credence to a diagnosis of coexisting iron deficiency anemia. Only after her Still's disease was put in remission did her serum ferritin achieve the subnormal levels diagnostic for iron deficiency anemia.

Although iron metabolism is complicated and many details of the qualitative ferritin changes in inflammatory disease are still unknown and we saw the patient only after her Still's disease and extreme hyperferritinemia resolved, a few observations may be noted that are relevant to the changes, their interaction with iron deficiency anemia, and how they might be modified in the extreme example of adult onset Still's disease.

Using the formula equating serum ferritin with iron stores in the normal state (1 *μ*g/L of serum ferritin = 8–10 mg of storage iron), our patient would be calculated to have 211 g of storage iron [14]. Yet this is clearly impossible even in persons with typical anemia of chronic inflammation, in whom the equation probably requires variable but modest modification. In Still's disease, even without iron deficiency, the equation obviously does not hold. In our patient with coexisting iron deficiency anemia and thus no available iron stores, it becomes clear that the extreme elevation of ferritin bears no relationship to body iron stores whatsoever. It is highly likely that her ferritin was virtually devoid of iron.

Ferritin normally consists of H and L isoforms. The H form is found in the heart, and the L form is found in the liver and formed during inflammation by the histiocyte-macrophage system. The L isoform holds less iron and is likely the form increased by cytokine-mediated ferritin release in Still's disease [11]. Ferritin glycosylation is also abnormal in patients with Still's disease. Glycosylation typically serves many roles, such as transport and protection of serum ferritin from proteolytic enzymes [13]. Healthy patients have 50–80% of their total serum ferritin glycosylated and this fraction falls to 20–50% in patients with inflammatory conditions [13, 15]. Whether our unique patient's serum ferritin has consisted even more disproportionately of the L isoform, was exclusively apoferritin, and had even more abnormal ferritin glycosylation than usual in Still's disease is unknown because we did not see her until her serum ferritin was nearly undetectable.

To our knowledge, this is the first documented case report of iron deficiency anemia in Still's disease with such an elevated ferritin. However, based on the usual predominance of Still's disease in women of menstruating age, one would wonder whether this is a more common phenomenon that is often undiagnosed. It is worthwhile for physicians to bear this in mind and look for clues such as disproportionate degree of microcytosis when evaluating patients with Still's disease.

## Figures and Tables

**Table 1:** Hematological data.

	Hospitalization					
	Day 0**	Day 6	Day 7	Day 10	6 months later^†^	10 months later
Hgb g/dL (12–16)*	10.3	9.2	8.9	9.3	9.2	11.9
MCV fl (80–94)*	67	68	68	70	70	81
Ferritin *μ*g/L (13–150)*	26,387	1,365	1,095	835	6.5	35
Iron *μ*g/dL (45–160)*	49				14	48
TIBC *μ*g/dL (240–450)*	215				306	244
Iron saturation % (20–55)*	23%				5%	20%

**Adult onset Still's disease was diagnosed and methylprednisolone treatment started.

^†^Treatment was started for iron deficiency anemia.

*Normal ranges are shown in parentheses.

**Table 2:** Ferritin levels in published surveys of inflammatory conditions.

Diagnosis	Ferritin (*μ*g/L)*	
	Range	Mean ± SD
Rheumatoid arthritis [6]		90 ± 123
Inflammatory bowel disease (*n* = 45) [7]	(34–650)	
SLE (*n* = 128) [6]		436 ± 1,186
Dermatomyositis/polymyositis (*n* = 9) [8]		1,097 ± 1,827
Acute hepatitis (*n* = 5) [8]		1,347 ± 861
Hemophagocytic syndrome (*n* = 7) [8]		8,485 ± 8,388
Autoimmune hepatitis (*n* = 1) [8]		11,262

Adult onset still's disease (*n* = 9) [9]	**(400–27,000)**	**13,910 ± 3,640**

*Mean ± SD.

*n* = Number of patients reported.
